# Sex-Specific Negative Association between Iron Intake and Cellular Aging Markers: Mediation Models Involving TNF*α*

**DOI:** 10.1155/2019/4935237

**Published:** 2019-11-11

**Authors:** Jie Yu, Haibin Liu, Shuli He, Pingping Li, Chunxiao Ma, Minglei Ma, Yiwen Liu, Lu Lv, Fan Ping, Huabing Zhang, Wei Li, Qi Sun, Lingling Xu, Yuxiu Li

**Affiliations:** ^1^Department of Endocrinology, Key Laboratory of Endocrinology, Ministry of Health, Peking Union Medical College Hospital, Beijing 100730, China; ^2^Department of Basic Physiology, The Health School Affiliated to Capital Medical University, China; ^3^Department of Nutrition, Peking Union Medical College Hospital, Beijing 100730, China; ^4^State Key Laboratory of Bioactive Substance and Function of Natural Medicines, Institute of Materia Medical Sciences and Peking Union Medical College, Beijing 100050, China; ^5^Diabetes Research Center of Chinese Academy of Medical Sciences, Beijing 100050, China

## Abstract

**Background:**

Given that the dysregulation of iron homeostasis leads to genomic instability, iron has been linked to cellular aging. However, epidemiological research on dietary iron intake and cellular aging markers is scarce. The aim of this study was to explore the relationship between dietary iron intake and cellular aging markers and to investigate whether tumor necrosis factor-*α* (TNF*α*) mediated this relationship.

**Methods:**

We conducted a cross-sectional analysis with a total of 467 subjects. Detailed dietary data were obtained using 24 h food recalls. Peripheral blood leukocyte telomere length (LTL) and mitochondrial DNA copy number (mtDNAcn) were assessed using real-time PCR assay. The association between dietary iron intake and cellular aging markers and TNF*α* and superoxide dismutase (SOD) was analyzed by Pearson correlation analysis and regression models adjusted by covariates. Simple mediation models were generated to examine whether TNF*α* mediated the association between iron intake and cellular aging markers using PROCESS macro Version 3.3.

**Results:**

The study population contained more women than men, but their basic demographic and metabolic characteristics did not differ. After adjusting for age, LTL was the same for men and women, while mtDNAcn was lower in men. Multiple linear regression adjusted for confounding factors found that iron intake was negatively associated with LTL only in women and negatively associated with mtDNAcn only in men. Moreover, iron intake was positively associated with TNF*α* in both women and men but positively associated with SOD only in men. Path modeling showed that TNF*α* significantly mediated the indirect detrimental effect of iron intake on LTL only in women; in men, mediation of the indirect effect of iron intake on mtDNAcn by TNF*α* did not reach significance.

**Conclusions:**

The study found sex-specific negative associations between dietary iron intake and cellular aging markers in that iron intake had deleterious effects on LTL attrition in women and mtDNAcn in men; only the former was partly mediated by TNF*α*. Consequently, when dietary iron intake and iron supplementation is recommended, the effects of iron imbalance on genomic stability and cellular aging markers must be considered.

## 1. Background

Telomeres are the cap structures at the ends of chromosomes in eukaryotic cells, rich in the noncoding repeat TTAGGG [1]. Telomeres shorten with each cell division and, to a certain extent, initiate cell senescence and apoptosis. As human cell telomeres shorten with age, peripheral blood leukocyte telomere length (LTL) has come to be considered a reliable marker of cellular aging and age-related diseases [2]. In addition to LTL, peripheral blood mitochondrial DNA copy numbers (mtDNAcn) also decrease with age and are found to be negatively correlated with the health status of elderly people, making mtDNAcn another reliable marker of aging and age-related disease [3].

Along with aging, inflammation and oxidative stress are the most studied factors to accelerate telomere shortening as well as mtDNA mutations and deletions [4, 5]. Furthermore, certain studies have found that diet is also closely related to cellular aging, as LTL has been negatively associated with the proinflammatory capacity of diet (as represented by the Dietary Inflammatory Index™ (DII®)), while positively correlated with the antioxidant capacity of the diet [6–8]. Our previous study also found that various dietary ingredients can have different effects on telomere length [9].

Iron is an essential element supplied mainly by dietary intake. It participates in a variety of physiological processes and must be maintained in a relatively narrow range to sustain metabolic homeostasis and genomic stability [10]. However, iron overload causes iron deposition in tissues and organs that plays a vital role in aging. Iron-fed rats exhibit increased iron levels in tissues and serum, increased serum transferrin saturation, and increased serum levels of TNF*α* and SOD [11]. The pathophysiological mechanism of the harmful effects of iron overload involves inflammation and oxidative stress caused by labile Fe, resulting in impaired stability and integrity of the genome [10]. The recommended nutrient intake (RNI) for dietary iron in Chinese residents is 9–20 mg/d for adults depending on age and sex. However, the current RNI for iron is mainly prescribed to prevent anemia; it does not take into account the genomic instability involved in the development of cellular aging and age-related diseases.

To date, there are few epidemiological studies on dietary iron intake and cellular aging markers, and the conclusions of those that do exist are inconsistent. An epidemiological survey involving 586 subjects 35–74 years of age found that subjects administered iron supplements had shorter telomeres than those without iron supplementation: 5121 ± 183 bps compared to 5583 ± 87 bps (29.0% difference; *p* = 0.007) [12]. Another cross-sectional study of 10 568 adults from the United States found that iron intake in the diet was positively correlated with telomere length in the peripheral blood [13]. Moreover, studies on the association between dietary iron and peripheral blood mitochondrial DNA copy number do not exist. Therefore, further population studies on dietary iron intake and genomic stability are necessary to understand the association and determine the most appropriate recommended intake range.

The aim of this population-based study was to explore the relationship between dietary iron intake and cellular aging markers, as well as to establish mediation models to explore whether TNF*α* mediated the relationship between them. Moreover, given that men and women differ in physical fitness and dietary habits, the study emphasized the sex differences involved in the association.

## 2. Methods

### 2.1. Study Population

The data used for this analysis were derived from a type 2 diabetes program in a Beijing suburb from 2014 to 2015 [9]. All subjects voluntarily submitted written informed consent, including a questionnaire about demographics, lifestyle, and disease and medication history. They also underwent detailed physical exams and a 2-hour oral glucose tolerance test (OGTT) after an overnight fast (>10 h).

**Figure 1:**
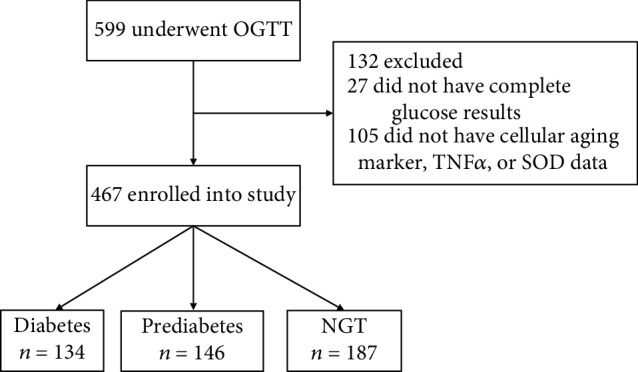
The flow diagram of the population-based study.

The 1999 World Health Organization (WHO) criteria were used to define the glucose tolerance status. Subjects that did not have cellular aging marker, TNF*α*, or SOD data were not included in the study, leaving a total of 467 individuals that were selected for the experiment (normal glucose tolerance (NGT; *n* = 187, including 129 women and 58 men), prediabetes (*n* = 146, including 91 women and 55 men), and diabetes mellitus (DM; *n* = 134, including 86 women and 48men)) (Figure 1). The study protocol was approved by the Ethics Committee of Peking Union Medical College Hospital.

### 2.2. Anthropometric Measurements

While subjects remained in a resting position for 15 minutes, a mercury sphygmomanometer was used to measure upper limb blood pressure; the values obtained from two measurements were averaged. Height and body weight were measured with subjects in light clothes and without shoes, and body mass index (BMI) was derived by dividing the body weight in kilograms by the square of the height in meters.

### 2.3. Biochemical Measurements

Hemoglobin (HbA1c) analysis was performed by high-performance liquid chromatography (HPLC; intra-assay coefficient of variation (CV) < 3%, interassay CV < 10%). An automated analyzer was used to determine low-density lipoprotein cholesterol (LDL-C), high-density lipoprotein cholesterol (HDL-C), triglyceride (TG), and cholesterol (TC) levels. TNF*α* and SOD were analyzed with kits in compliance with the manufacturer's protocols (Cloud-Clone Corp., Houston, TX, USA).

### 2.4. Measurement of LTL and mtDNAcn

Previous reports described peripheral blood LTL and mtDNAcn analysis in detail [9, 14]. In short, LTL was determined as the relative ratio of telomere-repeat copy number to single copy number (T/S) using the novel monochrome multiplex quantitative PCR protocol described by Cawthon [15]. The within-plate and between-plate CVs were 18% and 7%, respectively. The relative mtDNAcn was measured by real-time PCR and corrected by the simultaneous measurement of nuclear DNA. The average intra-assay and interassay CVs were 4.2% (range, 1.6%–9.8%) and 4.6% (range, 0.9%–7.8%), respectively.

### 2.5. Dietary Intake Assessment

Dietary information was collected using 24 h food recalls. The dietary data was reviewed by dietitians and entered into the nutrition calculation software (developed by researchers based on the Microsoft Office Access 2007 database). The food ingredient data was calculated using the China Food Composition Table (2004) database as a guide.

### 2.6. Statistical Analysis

Continuous data conforming to normal distributions were expressed as mean ± standard deviations (SDs) or standard errors (SEs); data not conforming to normal distributions were transformed. Categorical variables were expressed as percentages or ratios. A general linear model or chi-squared test was used to compare basic characteristics between males and females. Iron intake mg per 1000 kcal energy was also calculated.

Correlations between iron intake and cellular aging markers, TNF*α*, and SOD were explored using Spearman correlation analysis and multiple linear regression. In correlation analysis, iron intake was expressed as mg per 1000 kcal calories. In multiple linear regression, iron intake was also expressed as mg per 1000 kcal calories, and the B coefficients and 95% confidence intervals (CIs) as per 1SD increment of iron intake were calculated. Model 1 was age-adjusted, while model 2 was adjusted by age as well as hypertension status, BMI, and HbA1c, LnTG, HDL-C, and carbohydrate proportions.

To investigate whether TNF*α* was involved in the relationship between iron intake and cellular aging markers and whether mtDNAcn mediated the relationship between iron intake and SOD, PROCESS macro Version 3.3 [16] was used to generate simple mediation models using ordinary least squares. Mediation hypotheses were tested via a bias-corrected bootstrap method with 5000 samples to calculate confidence intervals (95%). Significance was achieved when an indirect effect was observed and zero was not included in confidence intervals. Iron intake was expressed as mg per 1000 kcal calories, and models were adjusted by age as well as hypertension status, BMI, and HbA1c, LnTG, and HDL-C. SPSS Version 25.0 (IBM Corp., Armonk, NY, USA) was used to perform all statistical analyses. All *p* values are two-tailed, with statistical significance determined when *p* < 0.05.

## 3. Results

### 3.1. Basic Characteristics of the Study Population

Table 1 shows the basic characteristics of the study population. More than half of the study population were women, with no difference in age between the two groups. The serum TNF*α* level was higher in women (borderline significant) and the serum SOD level was not different between the two groups. The peripheral blood mtDNAcn was significantly lower in men, but the LTL was not different between women and men. Men had greater dietary iron intake than women; conversely, this became slightly lower than women when adjusted for total daily energy intake or when iron intake was expressed as mg per 1000 kilocalories, although there was no statistical difference. There were no significant differences in BMI, blood glucose, blood lipids, or blood pressure between the two groups (Table 1).

### 3.2. Correlations between Iron Intake with Cellular Aging Markers, TNF*α*, and SOD

**Figure 2:**
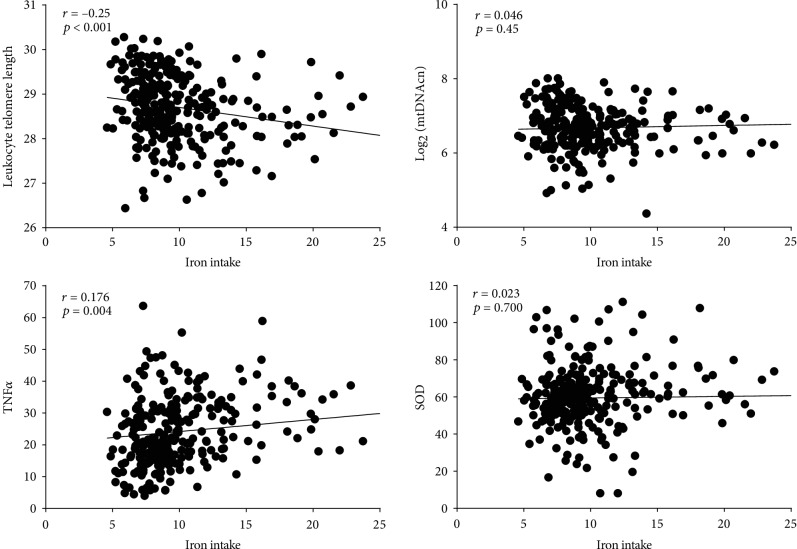
Correlation dot plots of iron intake and cellular aging markers, TNF*α*, and SOD in women. The abscissas are iron intake and the ordinates are LTL, mtDNAcn, TNF*α*, or SOD.

**Figure 3:**
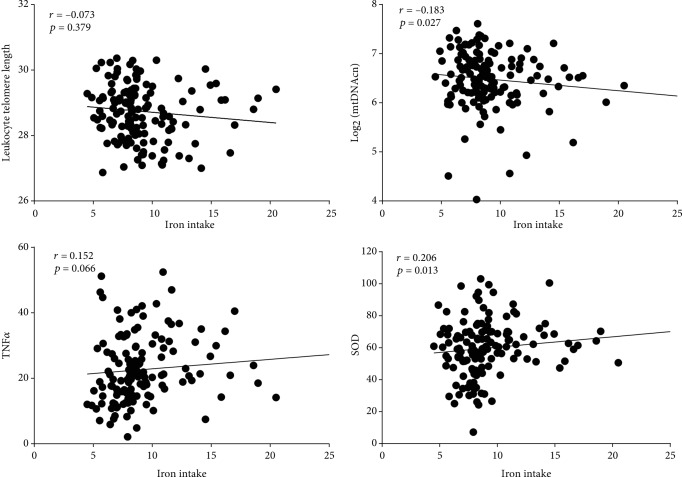
Correlation dot plots of iron intake and cellular aging markers, TNF*α*, and SOD in men. The abscissas are iron intake and the ordinates are LTL, mtDNAcn, TNF*α*, or SOD.

As is shown in Table 2, iron intake was negatively correlated with LTL (*r* = −0.25, *p* < 0.001), positively correlated with the serum TNF*α* level (*r* = 0.176, *p* = 0.004), but not correlated with mtDNAcn and serum SOD levels in women (Table 2, Figure 2). However, in men, iron intake was significantly negatively correlated with mtDNA rather than LTL and positively correlated with serum TNF*α* and SOD levels, with only the latter achieving statistical differences (Table 2, Figure 3).

### 3.3. Regression Analysis of Iron Intake and Cellular Aging Markers, TNF*α*, and SOD

Table 3 shows the coefficients of multiple linear regression analysis of iron intake and cellular aging markers, TNF*α*, and SOD. After adjusting for age, hypertension status, BMI, and HbA1c, LnTG, HDL-C, and carbohydrate proportion, iron intake was negatively associated with LTL only in women (*B* = −0.196, 95% CI: −0.296 to −0.096), while negatively associated with mtDNAcn only in men (*B* = −0.114, 95% CI: −0.223 to −0.005).

As for the inflammation marker, serum TNF*α* concentration was significantly positively associated with iron intake in both women and men (*B* = 1.535, 95% CI: 0.264 to 2.807, and *B* = 1.947, 95% CI: 0.151 to 3.742). In regard to the oxidative stress marker, there was a significantly positive association between iron intake and SOD only in men (*B* = 4.238, 95% CI: 1.033 to 7.444).

### 3.4. Mediation Models to Determine the Associations between Iron Intake and Cellular Aging Markers, TNF*α*, and SOD

**Figure 4:**
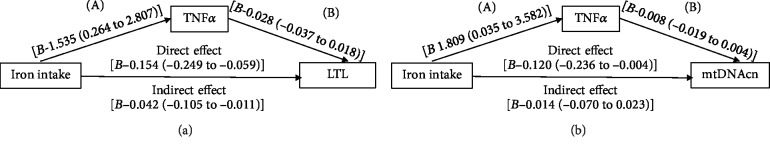
Path models of associations between iron intake, TNF*α*, and cellular aging markers. (a) The *p* value for regression (A) was 0.0182, for regression (B) was <0.001, and for direct effect was 0.0016. (b) The *p* value for regression (A) was 0.0457, for regression (B) was 0.1848, and for direct effect was 0.0429. As for the indirect effect, significance was achieved when zero was not included in confidence intervals and did not have a *p* value. Models were adjusted for age, hypertension status, BMI, and HbA1c, LnTG, HDL-C, and carbohydrate proportion.

In women, iron intake had direct negative effects on LTL (*B* −0.154, 95% CI −0.249 to −0.059); TNF*α* significantly mediated the indirect effect of iron intake on LTL (*B* −0.042, 95% CI −0.105 to −0.011) (Figure 4(a)). In men, iron intake also had direct negative effects on mtDNAcn (*B* −0.120, 95% CI −0.236 to −0.004), but the mediation of these indirect effects by TNF*α* did not reach statistical significance (Figure 4(b)).

**Figure 5:**
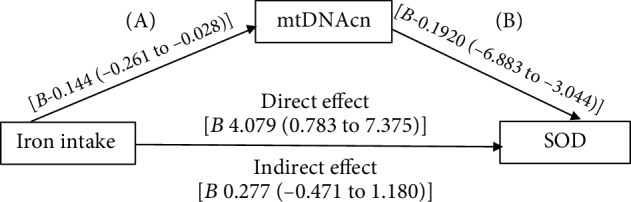
Path model of association between iron intake, mtDNAcn, and SOD. In Figure 4, the *p* value for regression (A) was 0.0153, for regression (B) was 0.4453, and for direct effect was 0.0157. As for the indirect effect, significance was achieved when zero was not included in confidence intervals and did not have a *p* value. Models was adjusted for age, hypertension status, BMI, and HbA1c, LnTG, HDL-C, and carbohydrate proportion.

Given that mitochondrial dysfunction may lead to an imbalance in oxidative stress, we further explored whether a decrease in mtDNAcn was associated with an increase in serum SOD concentration. As Figure 5 depicts, we did not find significant indirect effects of iron intake on SOD mediated by a decrease in mtDNAcn.

## 4. Discussion

The main findings of the study were that dietary iron intake was negatively associated with peripheral blood cellular aging markers, with sex differences. In women, iron intake was only found to be significantly negatively associated with LTL, whereas in men, iron intake was only found to be negatively associated with mtDNAcn. Furthermore, TNF*α* partially mediates the detrimental effect of iron intake on telomere shortening only in women.

Although evidence regarding the association between dietary iron and LTL is rare, our results are consistent with a previous study by Xu and colleagues showing that women administered iron supplements had shorter LTL than women without iron supplementation [12]. Moreover, both experiments and epidemiological studies have determined that iron overload is associated with telomere homeostasis [17–20]. A cross-sectional study of 1174 subjects 50–79 years of age found that serum transferrin saturation was inversely correlated with telomere length in the peripheral blood; furthermore, not only subjects with iron overload but also those with normal-high serum transferrin saturation values had shorter peripheral telomeres than subjects with normal-low serum values [18]. The study also found sex differences involved in these relationships in that normally high values of transferrin saturation are associated with shorter telomeres only in women, at least with any statistical significance [18]. Another cross-sectional study of 7336 adults over the age of 20 in the United States found that high ferritin levels were negatively correlated with telomere length only in women with statistical significance (*p* = 0.003), although there was no statistical difference in sex interaction (*p* for interaction = 0.12) [17].

To explore the mechanism underlying the detrimental effect of iron intake on telomere attrition, a path model was generated that found that the serum TNF*α* level partly mediated the association. On the one hand, a cross-sectional study of 1962 elderly people 70-79 years of age found that elevated serum TNF*α* was positively associated with increased risk of telomere shortening [21] and another cross-sectional study of 840 patients with cardiovascular disease found that polymorphisms in certain TNF loci were associated with elevated hypersensitive C-reactive protein and shortened telomere length in the peripheral blood [22]. On the other hand, basic experiments have determined that high iron ingestion increases the expression of TNF*α* in rat liver [23], and iron overload induces macrophage polarization imbalance, leading to increased expression of M1 macrophage-associated factors such as TNF*α* [24]. Therefore, our results are consistent with those reported in the literature and are convincing in terms of the mechanism of action we determined.

In regard to mtDNAcn and iron intake, their reverse association only reached statistical significance in men. Previous studies have also reported the sex difference in that the mitochondrial DNA copy number was found to decrease with age only in men [25]. While no direct evidence regarding the association between iron intake and mtDNAcn exists in the literature, iron has long been closely related to mitochondria [26]. Mitochondria are the centers of iron utilization and accumulation. Both iron deficiency and overload may affect the structure and function of mitochondria. Clinical evidence has manifested that mtDNA copy number is negatively correlated with transferrin in the cerebrospinal fluid of HIV-infected adult patients [27]. As mentioned earlier, increased iron intake was associated with elevated TNF*α*, which is a proinflammatory pathway leading to cellular inflammation and apoptosis [28] that has been associated with mitochondrial DNA damage. Basic research has established that TNF*α* acts directly on mitochondria through the TNF*α* receptor and that TNF*α* signaling causes *PGC1a* hypermethylation, resulting in decreased expression of PGC1a, decreased mitochondrial biogenesis, and decreased mitochondrial content [29]. Furthermore, patients with fibromyalgia and severe diabetic retinopathy have reduced mitochondrial DNA and elevated TNF*α* in their peripheral blood compared to control groups [30, 31]. However, we did not find a significant mediating effect of TNF*α* on the inverse association between iron intake and mtDNAcn, which is probably attributed to the relatively small sample size and insufficient test performance in this study.

Ultimately, we also found an inverse association between iron intake and SOD in men. Iron overload has been associated with increased oxidative stress [32]. Our results were consistent with experimental research that shows iron-fed rats have increased serum levels of SOD [11]. In order to further explore whether mitochondria participated in the association between iron intake and SOD, the mediation model was generated; it found no significant indirect effect of decreased mtDNAcn. Studies with larger sample sizes as well as mechanistic studies are necessary to further clarify these relationships.

### 4.1. Strengths and Limitations

This research presented some advantages and disadvantages. The first advantage was that the study consisted of subjects whose glucose metabolism status was available and continuous (from NGT to diabetes), making conclusions representative; the second advantage was that the detailed dietary data made it possible for the study to make up for the gaps in the association between iron intake and cellular aging markers. However, the disadvantage was that the study's sample size was relatively small and that the cross-sectional study did not yield a causal relationship. Moreover, although a single 24 h food recall was not that representative and had some limitations, our dietary composition investigation questionnaires were designed and assessed by clinical dieticians and the investigators were trained, so the validity and reliability of the dietary data can be guaranteed.

## 5. Conclusions

In conclusion, the study found a sex-specific negative association between dietary iron intake and cellular aging markers in that iron intake had deleterious effects on LTL attrition in women while mtDNAcn decreased in men; furthermore, the former was partly mediated by TNF*α*. When dietary iron intake and iron supplementation is recommended, the relationship between iron imbalance with genomic stability and cellular aging markers must be fully considered.

## Figures and Tables

**Table 1:** Basic characteristics of the study population.

Parameters	Women	Men	*p* value
*N* (%)	306 (65.5%)	161 (34.5%)	—
Age (years)	52.2 ± 0.7	53.9 ± 0.9	0.113
Body mass index (kg/m^2^)	26.5 ± 0.3	25.9 ± 0.4	0.169
LTL^a^	28.65 ± 0.05	28.74 ± 0.07	0.276
Log_2_ (mtDNAcn)^b^	6.65 ± 0.04	6.47 ± 0.05	0.01
Hypertension (*n*, %)	151 (49.7%)	82 (51.3%)	0.746
HbA1c (%)	5.90 ± 0.06	5.89 ± 0.09	0.942
LnTG (mmol/L)	0.37 ± 0.04	0.45 ± 0.05	0.205
HDL-C (mmol/L)	1.33 ± 0.02	1.27 ± 0.03	0.101
TNF*α* (pmol/mL)	24.46 ± 0.60	22.71 ± 0.82	0.084
SOD (U/mL)	2.49 ± 0.07	2.47 ± 0.09	0.845
Energy intake (kcal/d)	1414.04 ± 39.99	1885.51 ± 54.41	<0.001
Carbohydrate proportions	0.66 ± 0.01	0.65 ± 0.01	0.633
Iron intake (mg/d)^c^	14.75 ± 0.83	19.76 ± 1.13	<0.001
Iron intake (mg/d)^d^	16.96 ± 0.65	15.68 ± 0.90	0.258
Iron intake (mg/1000 kcal)^e^	10.46 ± 0.31	9.97 ± 0.43	0.350

LTL: leukocyte telomere length; mtDNAcn: mitochondrial DNA copy number; HbA1c: hemoglobin A1c; TG: triglyceride; HDL-C: high-density cholesterol lipoprotein; TNF*α*: tumor necrosis factor-*α*; SOD: superoxide dismutase. ^a^LTL was adjusted by age; ^b^mtDNAcn was adjusted by age; ^c^iron intake was not adjusted; ^d^iron intake was adjusted by energy intake; ^e^iron intake was calculated as per 1000 kcal energy intake. All variables are presented as means ± SEs.

**Table 2:** Correlation analysis between iron intake and cellular aging markers, TNF*α*, and SOD.

Parameters	Women		Men	
	*r*	*p* value	*r*	*p* value
LTL	−0.25	<0.001	−0.073	0.379
Log_2_ (mtDNAcn)	0.046	0.45	−0.183	0.027
TNF*α*	0.176	0.004	0.152	0.066
SOD	0.023	0.700	0.206	0.013

**Table 3:** *B* coefficients and 95% CIs of multiple linear regression analysis of iron intake and cellular aging markers, TNF*α*, and SOD.

Parameters	Women			Men		
	*B*	95% CI	*p*	*B*	95% CI	*p*
LTL						
Model 1	−0.213	(−0.308, −0.117)	<0.001	−0.068	(−0.213, 0.077)	0.357
Model 2	−0.196	(−0.296, -0.096)	<0.001	−0.102	(−0.251, 0.047)	0.177
Log_2_ (mtDNAcn)						
Model 1	0.024	(−0.060, 0.109)	0.571	−0.123	(-0.231, −0.015)	0.026
Model 2	0.054	(−0.032, 1.141)	0.217	−0.114	(−0.223, −0.005)	0.04
TNF*α*						
Model 1	1.944	(0.694, 3.193)	0.002	1.856	(0.137, 3.575)	0.035
Model 2	1.535	(0.264, 2.807)	0.018	1.947	(0.151, 3.742)	0.034
SOD						
Model 1	0.542	(−1.991, 3.067)	0.674	4.108	(1.266, 6.950)	0.005
Model 2	0.995	(−1.469, 3.459)	0.427	4.238	(1.033, 7.444)	0.01

Model 1 only adjusted for age; model 2 adjusted for age, hypertension status, BMI, and HbA1c, LnTG, HDL-C, and carbohydrate proportion.

## Data Availability

All the data generated or analyzed during this study are included in the article.

## Abbreviations

LTL: Leukocyte telomere length
OGTT: Oral glucose tolerance test
PCR: Polymerase chain reaction
BMI: Body mass index
BP: Blood pressure
Wt: Weight
Ht: Height
HbA1c: Hemoglobin
TG: Triglyceride
HDL-C: High-density lipoprotein cholesterol
SD: Standard deviation
TNF*α*: Tumor necrosis factor-*α*
SOD: Superoxide dismutase
NGT: Normal glucose tolerance
DM: Diabetes mellitus
CV: Coefficient of variation
ELISA: Enzyme linked immunosorbent assay
CI: Confidence interval.
