# Erratum to “Sex-Specific Negative Association between Iron Intake and Cellular Aging Markers: Mediation Models Involving TNF*α*”

**DOI:** 10.1155/2021/9875056

**Published:** 2021-08-30

**Authors:** Jie Yu, Haibin Liu, Shuli He, Pingping Li, Chunxiao Ma, Minglei Ma, Yiwen Liu, Lu Lv, Fan Ping, Huabing Zhang, Wei Li, Qi Sun, Lingling Xu, Yuxiu Li

**Affiliations:** ^1^Department of Endocrinology, Key Laboratory of Endocrinology, Ministry of Health, Peking Union Medical College Hospital, Beijing 100730, China; ^2^Department of Basic Physiology, The Health School Affiliated to Capital Medical University, China; ^3^Department of Nutrition, Peking Union Medical College Hospital, Beijing 100730, China; ^4^State Key Laboratory of Bioactive Substance and Function of Natural Medicines, Institute of Materia Medical Sciences and Peking Union Medical College, Beijing 100050, China; ^5^Diabetes Research Center of Chinese Academy of Medical Sciences, Beijing 100050, China

In the article titled “Sex-Specific Negative Association between Iron Intake and Cellular Aging Markers: Mediation Models Involving TNF*α*” [1], there were errors in Figures 4 and 5 and in Authors' Contributions.

Figure 4(a) should show “1.535” instead of “-1.535” and “-0.018” instead of “0.018.” The corrected figure is shown below and listed as Figure 1.

Figure 5 should show “-1.920” instead of “-0.1920” and “3.044” instead of “-3.044,” and the caption should state “In Figure 5” instead of “In Figure 4.” The corrected figure and corrected caption are shown below and listed as Figure 2.

There was an error in Authors' Contributions, where some of the contributions were listed incorrectly. The corrected section appears below:

## Figures and Tables

**Figure 1:**
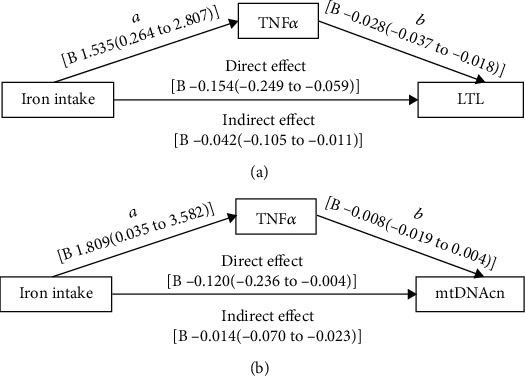
Path models of associations between iron intake, TNF*α*, and cellular aging markers. (a) The *p* value for regression (A) was 0.0182, for regression (B) was <0.001, and for direct effect was 0.0016. (b) The *p* value for regression (A) was 0.0457, for regression (B) was 0.1848, and for direct effect was 0.0429. As for the indirect effect, significance was achieved when zero was not included in confidence intervals and did not have a *p* value. Models were adjusted for age, hypertension status, BMI, and HbA1c, LnTG, HDL-C, and carbohydrate proportion.

**Figure 2:**
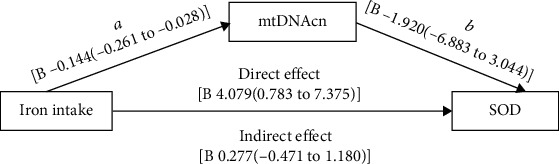
Path model of association between iron intake, mtDNAcn, and SOD. In Figure 5, the *p* value for regression (a) was 0.0153, for regression (b) was 0.4453, and for direct effect was 0.0157. As for the indirect effect, significance was achieved when zero was not included in confidence intervals and did not have a *p* value. Models were adjusted for age, hypertension status, BMI, and HbA1c, LnTG, HDL-C, and carbohydrate proportion.
